# Alteration of salivary *Streptococcus* is associated with statin therapy in older adults: a cohort study

**DOI:** 10.3389/fphar.2025.1455753

**Published:** 2025-04-07

**Authors:** Daisuke Hisamatsu, Yusuke Ogata, Wataru Suda, Yo Mabuchi, Yuna Naraoka, Taku Yamato, Akimi Ikeba, Kyoko Kumagai, Masahira Hattori, Chihiro Akazawa

**Affiliations:** ^1^ Intractable Disease Research Center, Juntendo University Graduate School of Medicine, Tokyo, Japan; ^2^ Laboratory for Symbiotic Microbiome Sciences, RIKEN Center for Integrative Medical Sciences, Yokohama, Japan

**Keywords:** common prescription drug, confounder, obesity, older adults, salivary microbiome, statin, *Streptococcus*

## Abstract

**Background:**

Salivary microbiome alterations are associated with chronic diseases, such as cardiovascular disease, diabetes, and dementia. These chronic diseases often coexist in older adults, leading to polypharmacy. This situation complicates the relationship between systemic diseases and salivary microbiome dysbiosis. Previous studies have demonstrated the association of the human gut microbiome with common prescription drug use, including polypharmacy. However, a comprehensive analysis of the salivary microbiome and prescription drugs is yet to be conducted in older adults. Therefore, in this study, we performed a multivariate analysis to investigate the relationship between salivary microbiomes and host variables, including prescribed drugs, cognitive function, and oral health, in Japanese older adults with different disease backgrounds.

**Methods:**

We enrolled non-hospitalised 82 older adults aged ≥70 years from a Japanese village community, and collected metadata, including age, sex, body mass index, cognitive function, oral health, alcohol consumption, smoking, and common prescription drug information. We performed multivariate analyses and functional predictions on the salivary microbiome based on 16S ribosomal RNA gene amplicon sequencing, including the metadata as potential confounders.

**Results:**

We observed a relationship between the human salivary microbiome and prescribed drug use in Japanese older adults with a heterogeneous background of comorbidities. The effects of several prescribed drugs, such as statins, proton pump inhibitors, and transporter/symporter inhibitors, on the salivary microbiome diversity were more prominent than those of host variables, including age, sex, and oral health. Notably, statin use was strongly correlated with a decrease in the *Streptococcus* abundance. Furthermore, statin intensity and obesity may be associated with altering the salivary microbiome, including functional predictions for vitamin biosynthesis and purine nucleotide degradation pathways in statin users.

**Conclusion:**

Our multivariate analysis, adjusted for prescribed drug use and non-use, revealed the drug-specific alteration of salivary microbiome composition in Japanese older adults with comorbidities. To our knowledge, this study is the first to described the association of common prescription drug use with salivary microbiome alterations in older adults. Our findings indicated that prescribed drug use is a key factor in understanding the link between salivary microbiome changes and systemic diseases in older adults.

## Introduction

The increasing prevalence of obesity in older adults is associated with a higher risk of cardiovascular diseases (hypertension and arrhythmia), dyslipidaemia, type 2 diabetes, and dementia (Mattiuzzi and Lippi, 2020). Previous studies have shown the evident association of obesity, including obesity-related comorbidities (inflammatory bowel diseases and atherosclerosis), with gut microbiome dysbiosis (Duvallet et al., 2017; Jie et al., 2017; Ley et al., 2006; Turnbaugh et al., 2006). Recently, gut microbiome alterations have been shown to be associated with transitions to Western diets, leading to the development of obesity, metabolic disease, and colorectal cancer in South Africa and Asia, including Japan (Chiba et al., 2021; Kolodziejczyk et al., 2019; Ramaboli et al., 2024; Yoshimatsu et al., 2021). However, research on the compositional change of individual bacteria is varied due to numerous confounding factors that affect the microbial structure (Escobar et al., 2014; Nishijima et al., 2016). In forensic science, human saliva, including the host genome, metabolite, and microbiome, is known as a promising application for inferring *postmortem* and individual characteristics such as dietary habits (Adserias-Garriga et al., 2017; Kamodyová et al., 2013; Sun et al., 2024). The relationship between the salivary or oral microbiome and host physiology, including systemic diseases and circadian rhythms, is also gaining attention (Said et al., 2014; Takayasu et al., 2017; Zhang et al., 2015). There is increasing interesting evidence that oral bacteria, including periodontitis-related pathobiont, translocate to the gut, exacerbating intestinal inflammation and the pathology of systemic diseases (Li et al., 2022b; Nagao et al., 2022; Yamazaki et al., 2021). Previous studies have revealed reductions in the salivary microbiome diversity in people with obesity (Shaalan et al., 2022; Wu et al., 2018). Further, the salivary microbiome composition was altered in individuals who underwent obesity surgery; however, these compositional observations were heterogeneous (Dzunkova et al., 2020). Numerous confounders in the salivary microbiome may lead to inter-individual variability. Therefore, the contribution of the salivary microbiome to the pathogenesis of obesity remains unclear.

Older adults with chronic diseases, including obesity, tend to practice polypharmacy (Tsoi et al., 2014). Polypharmacy has been associated with increased rates of hospitalisation and mortality in older adults (Chang et al., 2020). The most common prescription drugs are associated with cardiovascular diseases, such as anti-hypertensives (calcium channel blockers) and anti-hyperlipidaemics (statins), and gastrointestinal disorders, such as gastric acid suppressants (proton pump inhibitors [PPIs]), in older adult populations, including Japan (Ishizaki et al., 2020; Tsoi et al., 2014). Statins, also known as 3-hydroxy-3-methylglutaryl coenzyme A (HMG-CoA) reductase inhibitors, are first-line drugs for dyslipidaemia; other anti-dyslipidaemic drugs are cholesterol-absorption inhibitors and fibrates (Grundy, 2006). Recent studies at the population level have revealed a strong relationship between gut microbiome alterations and therapeutic drug use, including PPIs and statins (Falony et al., 2016; Jackson et al., 2018; Nagata et al., 2022; Vich Vila et al., 2020). However, the relationship between the salivary microbiome and prescribed drugs remains unclear. A cohort study of the association of the salivary microbiome with common prescription drug use, including polypharmacy, in older adults is required to understand the connection between salivary microbiome alteration and the development of systemic diseases.

Therefore, we performed a multivariate analysis to investigate the relationship between salivary microbiomes and host variables, including prescribed drugs, cognitive function, and oral health, in Japanese older adults with different disease backgrounds. Our findings may provide new insights into the association of systemic diseases with the human salivary microbiome, particularly concerning prescribed drug use.

## Materials and methods

### Study design

We enrolled 82 participants from a Japanese village volunteer cohort consisting of older adults aged ≥70 years. The following metadata were collected: age, sex, body mass index (BMI), prescribed drug information, cognitive function scores [Mini-Mental State Examination (MMSE) and revised Hasegawa Dementia Scale (HDS-R) scores], and questionnaire responses (frequency of drinking alcohol, smoking status, and oral health information). The participants were diagnosed with cognitive impairment using cognitive function test scores and grouped into normal (MMSE ≥28 and HDS-R ≥21) and impairment (24≤ MMSE ≤27 and HDS-R ≥21) groups. We further analysed 71 participants with complete prescription drug information. Inclusion criteria were (1) 70 years of age or older, (2) capable of independently providing written informed consent, and (3) capable of everyday conversation in Japanese. Exclusion criteria were (1) hospitalised patients, (2) the presence of visible oral and jaw lesions, and (3) the use of antibiotics within 3 months before sampling following previous studies (Huse et al., 2008; Rashid et al., 2015).

### Classification of prescribed drugs

The 153 prescribed drugs were classified into 26 categories based on their mode of action (Supplementary Table S1). In this study, the drug categories were not divided based on disease specificity (anti-diabetic drugs) due to the difficulty in distinguishing between the effects of the disease and those of drug use on the microbiome, as suggested by Vich Vila et al. (Vich Vila et al., 2020). Briefly, drug classes share a common molecular mode of action modulating the activity of a specific biological target based on a previous review (Imming et al., 2006). In the case of enzymes, these activities include activators or inhibitors [e.g., angiotensin-converting enzyme (ACE) inhibitors]. Receptor-targeting activities include agonists [e.g., benzodiazepine (BZD) receptor agonists] or antagonists (e.g., adrenergic receptor antagonists). Ion channel-targeting activities include openers (e.g., potassium channel openers) or blockers (e.g., sodium channel blockers). We first divided the site of action into the central nervous system (CNS) or peripheral tissues. We further categorised based on the type of molecular targets [e.g., arachidonate cascade, renin-angiotensin-aldosterone system (RAAS), and statins]. Exclusion drugs were eye drops, nasal drops, and topical steroids in this study. Statin intensity was determined by type and dose following a previous study (Stone et al., 2014).

### Questionnaire

Dental treatment was divided into two scales: currently undergoing treatment (including routine examinations) or not. The usage of dentures was divided into two scales: users and non-users. Following a previous study (Blaustein et al., 2021; Pyysalo et al., 2019), the toothbrushing frequency was on a scale of once or, twice or more. Oral dryness was self-reported as a daily feeling or not. The smoking frequency was on a scale of never or smoking. The frequency of alcohol consumption was on a scale of regulatory (three or more times per week) or not, following a previous study (Vujkovic-Cvijin et al., 2020).

### Saliva collection and 16S ribosomal RNA gene amplicon sequencing

All saliva samples were collected during the daytime (9:30–16:00), following a previous study (Iwasawa et al., 2018). The samples were collected using a sterilised sputum container (DE 2000; Eiken Chemical Co., Tokyo, Japan), kept at 4°C until immediately frozen with liquid nitrogen (within 8 h after sampling), and stored at −80°C until DNA extraction. Sampling, freezing, and DNA extraction through enzymatic lysis were performed following previous studies (Iwasawa et al., 2018; Said et al., 2014). The 16S ribosomal RNA (rRNA) gene amplicons (V1-V2 region) were obtained by performing polymerase chain reaction (PCR) using the following primers, containing the Illumina Nextera adapter sequence and a unique 8-bp index sequence for each sample (indicated by xxxxxxxx): forward 27Fmod (5′-AATGATACGGCGACCACCGAGATCTACACxxxxxxxxACACTCTCTTTCCCTACACGACGCTCTTCCGATCTagrgtttgatymtggctcag-3′) and reverse 338R (5′-CAAGCAGAAGACGGCATACGAGATxxxxxxxxGTGACTGGAGTTCAGACGTGTGCTCTTCC GAT​CTt​gct​gcc​tcc​cgt​agg​agt-3′). The thermal cycling of the PCR was performed on a 9700 PCR system (Life Technologies, Carlsbad, CA, United States) using Ex Taq polymerase (Takara Bio, Tokyo, Japan) with the following conditions: initial denaturation at 96°C for 2 min; 25 cycles of denaturation at 96°C for 30 s, annealing at 55°C for 45 s, extension at 72°C for 1 min, and final extension at 72°C. The 16S rRNA gene amplicons were purified using AMPure XP magnetic purification beads (Beckman Coulter, Brea, CA, United States) and quantified using the Quant-iT PicoGreen dsDNA Assay Kit (Life Technologies, Japan). The amplicon pools were sequenced on the Illumina MiSeq Platform (2 × 300 bp), following the manufacturer’s instructions.

### Data processing

The analysis pipeline for MiSeq-barcoded amplicon sequencing was conducted following previous studies (Aiyoshi et al., 2023; Li et al., 2022a). Primer sequences were trimmed from the paired-end 16S rRNA gene amplicon using Cutadapt v.4.1–1. To construct amplicon sequence variants (ASVs), the trimmed reads were uploaded to the DADA2 R package v.1.18.0, and possible chimeric reads were removed. We adopted 10,000 filter-passed reads per sample of high-quality reads and deposited them in the DDBJ/GenBank/EMBL database. The taxonomy assignment of ASVs was determined by similarity searches against the National Center for Biotechnology Information RefSeq database, downloaded on 8 January 2020, using the GLSEARCH programme.

### Microbiome and metadata analysis

We analysed microbiome diversity as previously described (Iwasawa et al., 2018). Unweighted UniFrac (i.e., measuring the difference between microbial communities based on the presence or absence of species without considering their abundance) or weighted UniFrac (i.e., considering both the presence/absence and the relative abundance of species) distance analyses were used to determine the dissimilarity (distance) between each pair of samples. Dissimilarity in the microbiome composition was visualised using principal coordinate analysis based on UniFrac distance analysis. Statistical significance was obtained through permutational multivariate analysis of variance, and *P*-values were adjusted using the Benjamini–Hochberg method. The alpha diversity (α-diversity) was calculated as a Shannon index using the vegan package (v2.6−4) of the statistical programming language R, version 4.0.3 (2020-10-10).

Stepwise redundancy analysis (RDA) was performed to evaluate the confounding variables that contributed to the microbiome composition using the ordiR2step function (default direction = both) in the vegan package (v2.6−4), as previously described (Nagata et al., 2022; Nishijima et al., 2022). Distance-based RDA was performed using the Bray–Curtis distance with 999 permutations, and the *P*-values were corrected using the Benjamini–Hochberg method. This analysis estimates the linear cumulative and individual effect size of all identified microbiome covariates. Individual adjusted *R* ^*2*^ refers to the explained variance when the most influenced variable’s adjusted *R* ^*2*^ is maximised in the first step involving all microbiome covariates.

Multivariate analysis of the microbial community was performed through the R package MaAsLin2 (v1.10.0) using generated linear and mixed models (default model: min abundance = 0.00; min prevalence = 0.10; max significance = 0.25; normalisation = TSS; transformation = LOG) (Mallick et al., 2021), as previously described (Nagata et al., 2023). For the model of statin users, fixed effects included prescribed drugs (statin), whereas random effects encompassed age, sex, and four prescribed drugs (CNS drugs, transporter/symporter inhibitors, PPIs, and bisphosphonates) affected by the stepwise RDA and diversity analyses.

Functional predictive analysis based on the 16S rRNA gene sequencing data was performed using PICRUSt2 (phylogenetic investigation of communities by reconstruction of unobserved states) v2.6.0 (Douglas et al., 2020). Raw ASV count data was imported and run through the PICRUSt2 pipeline with default parameters. Briefly, the aligned ASVs were placed into a reference tree and were then used to infer gene family copy numbers of each ASV. The abundances of Kyoto Encyclopedia of Genes and Genomes (KEGG) orthology terms, Enzyme Commission (EC) terms and MetaCyc pathway were obtained by PICRUSt2.

### Statistical analyses

All statistical analyses were performed using the statistical programming language R version 4.3.3 (2024-02-29). Statistical significance was determined using the Wilcoxon rank-sum test through the Benjamini–Hochberg method. The *Q*-value, FDR (false discovery rate), and *P*-values were set at <0.05.

### Study approval

The Ethics Committee of Juntendo University School of Medicine approved this study (approval number H19-0244). All participants provided written informed consent for study participation. The procedures in this study were performed following the principles of the Declaration of Helsinki.

## Results

### Prescribed drug use as a salivary microbiome confounder

**FIGURE 1:**
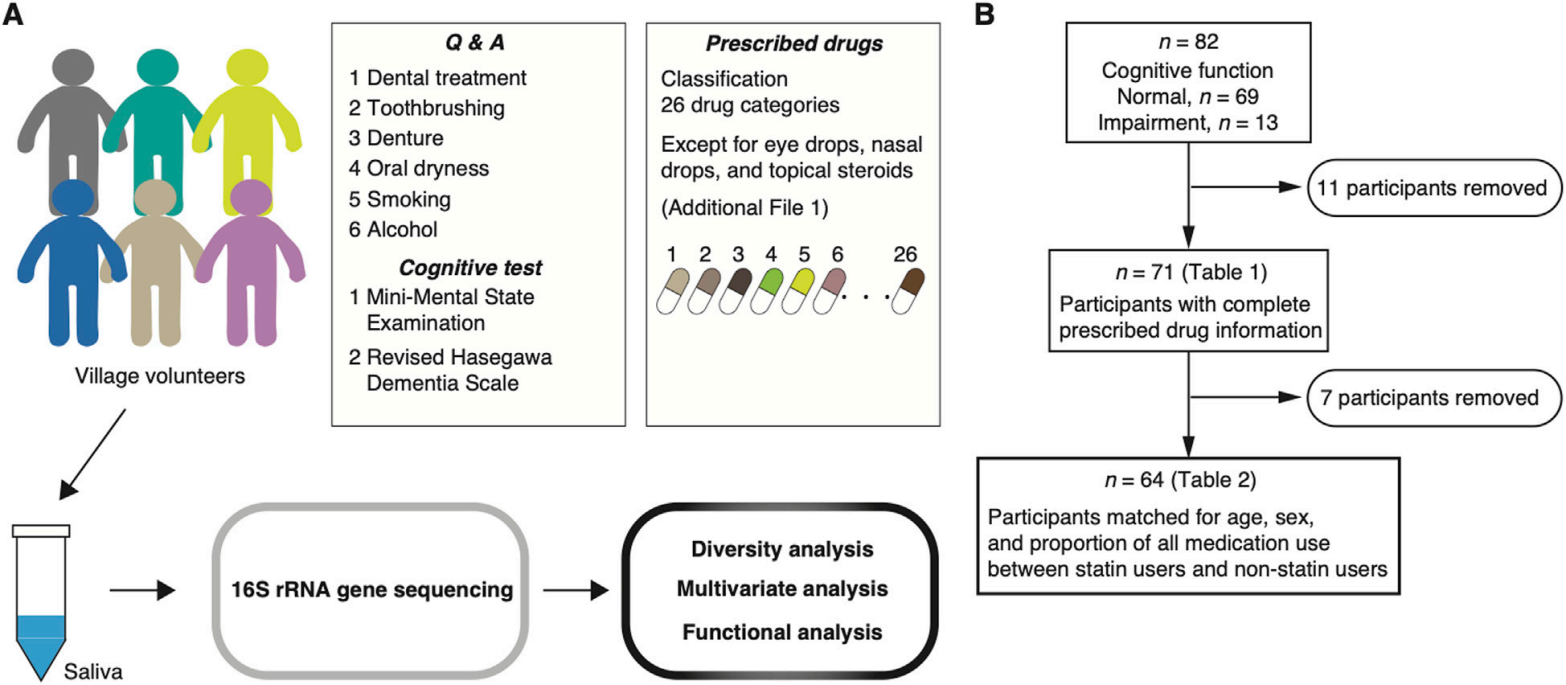
Study design. **(A, B)** Participants are recruited from a Japanese village volunteer cohort. The cohort comprises volunteers aged ≥70 years with diverse disease backgrounds enrolled at a municipal facility. Cognitive function tests and a questionnaire, including oral health and the frequency of smoking and alcohol consumption, are administered. Prescribed drug information is collected from a personal medication diary (the “Okusuri-techo”) and an interview. Diversity and multivariate analyses on the salivary microbiome are performed in participants with complete prescribed drug information (*n* = 71). To further evaluate the effect of statin use on the salivary microbiome, we selected participants who matched for age, sex, and the proportion of all medication use between non-statin and statin users (*n* = 64).

**FIGURE 2:**
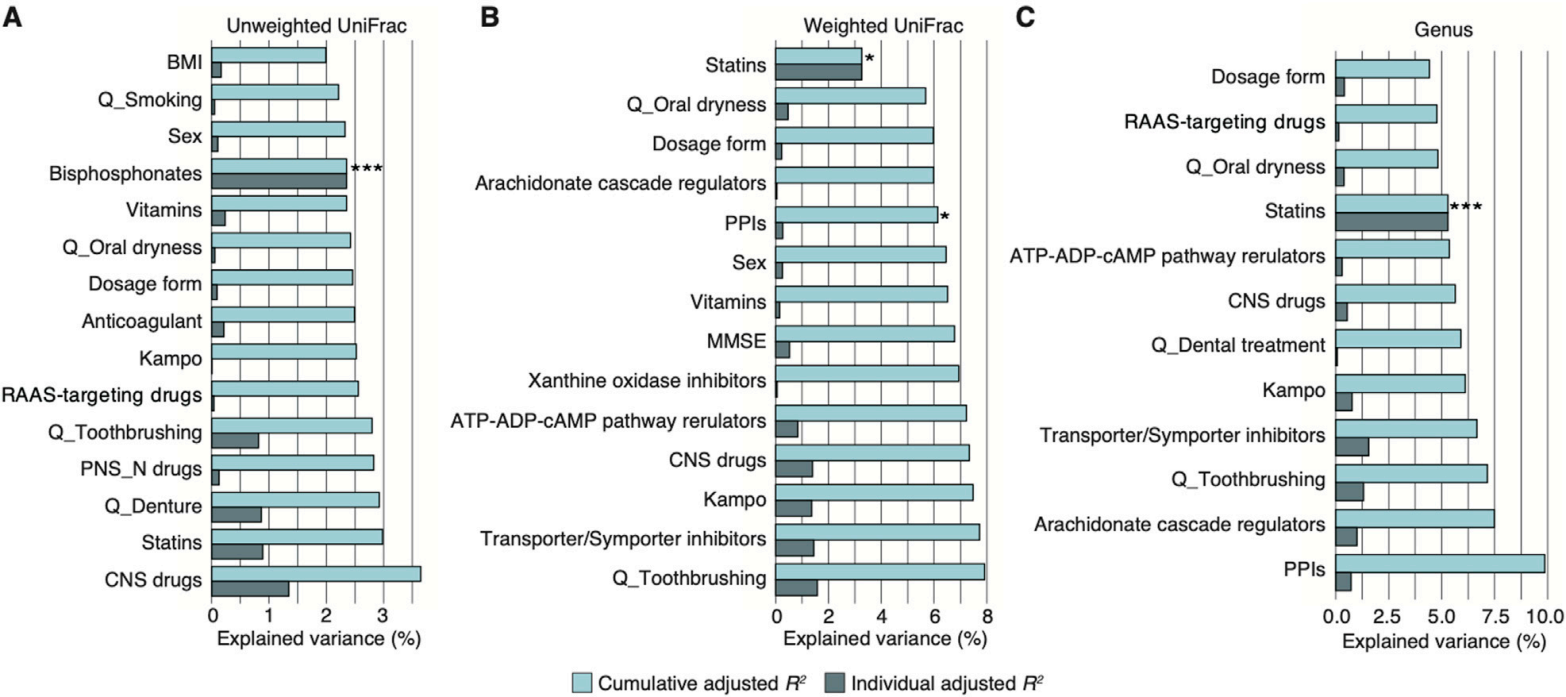
Salivary microbiome association with prescribed drug use in Japanese village volunteers with heterogenous disease backgrounds. **(A–C)** Individual and cumulative adjusted *R* ^*2*^ (explained variance) of covariates in stepwise redundancy analysis using unweighted **(A)** and weighted UniFrac distance **(B)** and at the genus level **(C)** in the village volunteer cohort (*n* = 71, *n* ≥ 3 per each drug category). The graph excludes negative values for individual adjusted *R* ^*2*^. *P < 0.05; ***P < 0.005. CNS, central nervous system; MMSE, Mini-Mental State Examination; PNS-N, peripheral nervous system via neurotransmitters; PPI, proton pump inhibitor; Q, questionnaire; RAAS, renin-angiotensin-aldosterone system.

The older adults in the study did not include patients with dementia, and their most commonly prescribed drugs were voltage-gated ion channel-targeting drugs (43.7%), including dihydropyridine calcium channel blockers and potassium channel openers and blockers, statins (38.0%), and RAAS-targeting drugs (28.2%), including ACE inhibitors and angiotensin receptor antagonists (Table 1; Supplementary Table S1). We initially performed a stepwise RDA based on 16S rRNA gene sequence data to evaluate how variables, including age, sex, cognitive function, oral health, and prescribed drugs, contributed to the salivary microbiome composition (Figure 1; Table 1). Notably, three drugs significantly contributed to the overall microbiome composition (cumulative adjusted *R* ^*2*^: Figures 2A–C; Supplementary Table S2): 1) unweighted UniFrac distance: bisphosphonates = 2.4% (*P* = 0.001); 2) weighted UniFrac distance: statins = 3.3% (*P* = 0.014) and PPIs = 6.1% (*P* = 0.015); and 3) at the genus level: statins = 5.3% (*P* = 0.004). No significant contributions to the salivary microbiome composition were observed in the host variables, including alcohol consumption frequency, smoking status, oral health (toothbrushing frequency, denture use, presence of oral dryness, and history of dental treatments), and cognitive function (Figures 2A–C). These results indicate that several prescribed drugs may be confounding factors for the salivary microbiome alteration in older adults with diverse comorbidities. Thus, alterations in prescribed drug use greatly influenced the salivary microbiome compared with changes associated with age, sex, oral health, and cognitive function.

**TABLE 1:** Number and percentage of prescribed drug users in our cohort.

Demographics	
Number of participants	82
Number of participants with complete prescribed drug information	71
Age (mean ± SD, years)	77.1 ± 4.8
Sex (% male)	18.3
MMSE (mean ± SD)	28.8 ± 1.8
HDS-R (mean ± SD)	28.6 ± 2.0
Number of users (%)	
CNS drugs	4 (5.6%)
PNS-N drugs	19 (26.8%)
PNS-V drugs	1 (1.4%)
Arachidonate cascade regulators	14 (19.7%)
Steroids	1 (1.4%)
RAAS-targeting drugs	20 (28.2%)
Voltage-gated ion channel-targeting drugs	31 (43.7%)
Transporter/Symporter inhibitors	9 (12.7%)
Isosorbide	1 (1.4%)
DPP-4 inhibitors	5 (7.0%)
Metformin	1 (1.4%)
Statins	27 (38.0%)
Ion-exchange resins	1 (1.4%)
Fibrate	2 (2.8%)
Anticoagulants	7 (9.9%)
ATP-ADP-cAMP pathway regulators	5 (7.0%)
Xanthine oxidase inhibitors	4 (5.6%)
PPIs	18 (25.4%)
Bronchodilator	1 (1.4%)
Thyroid hormone	1 (1.4%)
Bisphosphonates	4 (5.6%)
ERAAs	2 (2.8%)
Vitamins	7 (9.9%)
Cathartics	2 (2.8%)
Probiotics	3 (4.2%)
Kampo	5 (7.0%)

CNS, central nervous system; DPP4, dipeptidyl-peptidase 4; ERAAs, estrogen receptor agonists/antagonists; HDS-R, revised Hasegawa Dementia Scale score; MMSE, Mini-Mental State Examination; ns, not significant; PNS-N, peripheral nervous system via neurotransmitters; PNS-V, peripheral nervous system through voltage-gated ion channels drugs; PPIs, proton pump inhibitors; RAAS, renin-angiotensin-aldosterone system.

### Association of the salivary microbiome diversity with prescribed drug use

**FIGURE 3:**
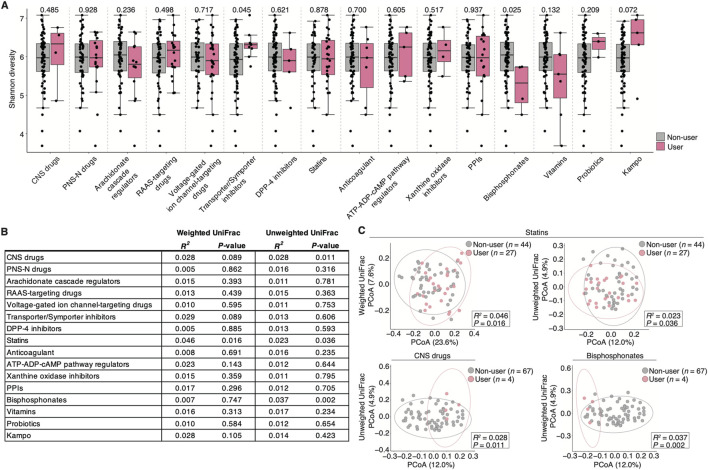
Salivary microbiome diversity alterations in prescribed Japanese drug users with heterogenous disease backgrounds. **(A)** Comparison of the alpha-diversity score (Shannon index) by each drug category (*n* = 71, *n* ≥ 3 per each drug category; see Table 1). Statistical significance is determined using the Wilcoxon rank-sum test (*P* < 0.05). Dots represent individual participants. **(B)** Permutational multivariate analysis of variance based on the weighted and unweighted UniFrac distance in each drug category (*n* = 71, *n* ≥ 3 per each drug category; see Table 1). **(C)** Weighted and unweighted UniFrac-PCoA in each drug category with a significant difference. The number of participants in each drug category is depicted in the figure. The *R* ^*2*^ and *P*-values are determined using permutational multivariate analysis of variance via the Benjamini–Hochberg method. Dots represent individual participants. CNS, central nervous system; DPP-4, dipeptidyl-peptidase 4; PPI, proton pump inhibitor; PNS-N, peripheral nervous system via neurotransmitters; RAAS, renin-angiotensin-aldosterone system.

We performed diversity analyses on each prescribed drug user to further evaluate the impact of the confounding prescribed drugs on the salivary microbiome. The species richness and evenness (α-diversity) were significantly higher in transporter/symporter inhibitor users but lower in bisphosphonate users compared with that in non-users (*P* = 0.045 and 0.025, respectively; Figure 3A). Furthermore, the inter-individual diversity (β-diversity) among users of prescribed drugs such as statins, CNS drugs, and bisphosphonates exhibited significant alterations compared with non-users (*R* ^*2*^; Figures 3B, C): 1) weighted UniFrac distance: statin = 0.046 (*P* = 0.016); and 2) unweighted UniFrac distance: statins = 0.023 (*P* = 0.036), CNS drugs = 0.028 (*P* = 0.011), and bisphosphonates = 0.037 (*P* = 0.002). However, the number of bisphosphonate and CNS drug users was small (*n* = 4 per drug category; Table 1). These results suggest that using several drugs may be associated with altering salivary microbiome diversity in older adults.

### Polypharmacy is not associated with salivary microbiome composition

The number of prescribed drugs was categorised into three groups (taking 0, one to five, and ≥6 drugs) based on previous studies (Kojima et al., 2012; Nagata et al., 2022; Vich Vila et al., 2020). The α-diversity tended to decline with increasing the number of drugs used (taking 0 vs. one to five groups, *P* = 0.333; taking 0 vs. ≥6 groups, *P* = 0.691; taking 1–5 vs. ≥6 groups, *P* = 0.333; Supplementary Figure S1A). No significant changes were found in the β-diversity among these groups (Supplementary Figure S1B). Moreover, we evaluated the correlation between the number of prescribed drugs and the bacterial abundance at the genus level. A Spearman’s correlation coefficient analysis revealed that the abundance of *Lachnoanaerobaculum* and *Gemella* increased under conditions of polypharmacy (*P* = 0.056 and 0.063, respectively; Supplementary Figure S1C). We also investigated the effect of the dosage form because the taste of oral disintegrating drugs is masked by sweeteners and flavouring agents (Reo and Fredrickson, 2002). The α- and β-diversities did not differ significantly according to dosage form (Supplementary Figure S2). These results suggest that the number of prescribed drugs and dosage form had less influence on the salivary microbiome diversity.

### Statin user-specific salivary microbiome alterations

**FIGURE 4:**
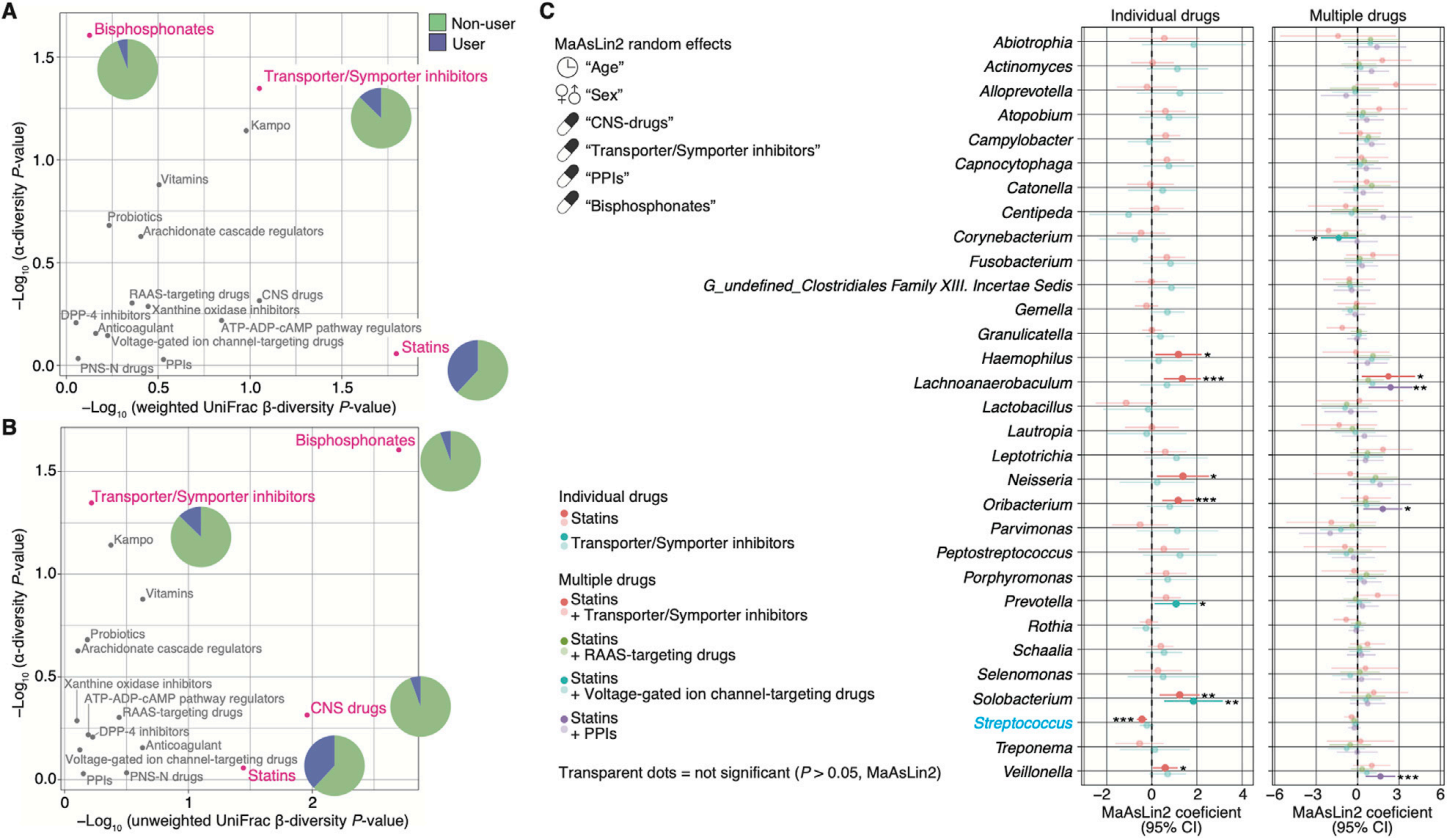
Alteration of microbial abundance depends on the prescribed drug used. **(A, B)** Graphs showing the effect size estimates based on diversity scores of each prescribed drug. Effect sizes captured as −log10 (*P*-value) of the alpha-diversity are shown on the X-axis, and as–log10 (*P*-value) of β-diversity based on weighted **(A)** and unweighted **(B)** UniFrac distances are shown on the Y-axis. Magenta dots represent drug categories with significant differences. Grey dots represent drug categories with no significant differences. The pie charts within the figure show the percentage of usage of the drug category with significant differences (also shown in Table 1). **(C)** Graphs show representative genera: the top 31 abundant genera with a relative mean abundance of >0.1%, enriched and depleted among participants using individual or combination of the prescribed drugs (*n* = 64; see Table 2). The number of multiple drug users is as follows: statin plus transporter/symporter inhibitors = 3, statin plus RAAS-targeting drugs = 10, statin plus voltage-gated ion channel-targeting drugs = 12, and statin plus PPIs = 9. Statistical significance and 95% confidence interval are determined using the MaAsLin2 package with age, sex, and the prescribed drugs contributing to the microbiome (CNS drugs, transporter/symporter inhibitors, PPIs, and bisphosphonates) as random effects (*P* < 0.05). *P < 0.05; **P < 0.01; ***P < 0.005. Confidence interval, CI; CNS, central nervous system; DPP-4, dipeptidyl-peptidase 4; PPI, proton pump inhibitor; PNS-N, peripheral nervous system via neurotransmitters; RAAS, renin-angiotensin-aldosterone system.

We applied diversities as an indicator to evaluate which prescribed drug use had a greater influence on the salivary microbiome. The use of bisphosphonates showed the largest impact on α- and β-diversity, but its usage was low at 5.6% (Figures 4A, B; Table 1). The effect of transporter/symporter inhibitors with a usage rate of 12.7% was observed in intra-individual diversity (Figures 4A, B; Table 1). However, statins had a low impact on intra-individual diversity but a large inter-individual difference between users and non-users, with a high usage rate of 38.0% (Figures 4A, B; Table 1). We then explored the salivary microbiome alteration specific to statin users because statin is one of the most common prescription drugs in older adults (Ishizaki et al., 2020; Tsoi et al., 2014). In addition, as transporter/symporter inhibitors include anti-dyslipidaemia drugs (cholesterol-absorption inhibitors; Supplementary Table S1), we investigated the interaction between statins and transporter/symporter inhibitors. The cohort was divided into statin users and non-users with all matched metadata, including prescribed drug use and oral health (Table 2). We performed a multivariate analysis considering age, sex, and the use of the four drugs (CNS drugs, transporter/symporter inhibitors, PPIs, and bisphosphonates) affected by the stepwise RDA and diversity analyses. We found that the abundance of *Lachnoanaerobaculum*, *Oribacterium*, *Solobacterium*, *Neisseria*, *Haemophilus*, and *Veillonella* increased significantly, whereas that of *Streptococcus* decreased significantly in statin users (*P* < 0.05; Figure 4C; Supplementary Table S3). The *Solobacterium* abundance increased significantly also in the transporter/symporter inhibitor users (*P* = 0.007; Figure 4C; Supplementary Table S3). The *Prevotella* abundance increased significantly only in the transporter/symporter inhibitor users (*P* = 0.031; Figure 4C; Supplementary Table S3). Notably, a significant increase was observed only for *Lachnoanaerobaculum* in users taking these two drugs (*P* = 0.027; Figure 4C; Supplementary Table S3). We further analysed the effect of combining statins and drugs with high usage rates, including voltage-gated ion channel-targeting drugs (50.0%), RAAS-targeting drugs (41.7%), and PPIs (37.5%; Table 2). No significant differences were observed in combinations with RAAS-targeting and voltage-gated ion channel-targeting drugs (Figure 4C). The abundance of *Veillonella*, *Lachnoanaerobaculum*, and *Oribacterium* increased significantly in PPI and statins-only users (*P* < 0.05; Figure 4C; Supplementary Table S3). These results suggest that the influence of statins on individual salivary bacteria is greater than that of cholesterol-absorption inhibitors among anti-dyslipidaemia drug users.

**TABLE 2:** Cohort demography between the statin-users and non-users.

	Non-statin user	Statin user	*P*-value
Number of participants	40	24	—
Age (mean ± SD, years)	76.8 ± 4.5	77.2 ± 4.8	0.893
Sex (% male)	20.0	16.7	ns
BMI (mean ± SD)	24.0 ± 3.7	22.9 ± 2.6	0.279
MMSE (mean ± SD)	29.2 ± 1.1	28.6 ± 2.3	0.502
HDS-R (mean ± SD)	28.9 ± 1.4	28.5 ± 2.6	0.790
Number of users (%)			
CNS drugs	3 (7.5%)	1 (4.2%)	ns
PNS-N drugs	9 (22.5%)	6 (25.0%)	ns
Arachidonate cascade regulators	6 (15.0%)	6 (25.0%)	ns
RAAS-targeting drugs	9 (22.5%)	10 (41.7%)	ns
Voltage-gated ion channel-targeting drugs	16 (40.0%)	12 (50.0%)	ns
Transporter/Symporter inhibitors	5 (12.5%)	3 (12.5%)	ns
DPP-4 inhibitors	4 (10.0%)	1 (4.2%)	ns
Anticoagulants	3 (7.5%)	1 (4.2%)	ns
ATP-ADP-cAMP pathway regulators	2 (5.0%)	3 (12.5%)	ns
Xanthine oxidase inhibitors	3 (7.5%)	1 (4.2%)	ns
PPIs	6 (15.0%)	9 (37.5%)	ns
Bisphosphonates	0 (0.0%)	2 (8.3%)	0.137
Vitamins	3 (7.5%)	3 (12.5%)	ns
Probiotics	2 (5.0%)	1 (4.2%)	ns
Kampo	2 (5.0%)	2 (8.3%)	ns
Number of positive answers (%)			
Q_Dental treatment	6 (15.0%)	8 (33.3%)	ns
Q_Toothblushing	once, 5 (12.5%) twice, 35 (87.5%)	once, 1 (4.2%) twice, 23 (95.8%)	ns
Q_Denture	20 (50.0%)	14 (58.3%)	ns
Q_Oral dryness	15 (37.5%)	4 (16.7%)	ns
Q_Smoking	3 (7.5%)	1 (4.2%)	ns
Q_Alcohol	5 (12.5%)	4 (16.7%)	ns

BMI, body mass index; CNS, central nervous system; DPP4, dipeptidyl-peptidase 4; HDS-R, revised Hasegawa Dementia Scale score; MMSE, Mini-Mental State Examination; ns, not significant; PNS-N, peripheral nervous system via neurotransmitters; PPIs, proton pump inhibitors; RAAS, renin-angiotensin-aldosterone system.

### *Streptococcus* is associated with obesity in statin users

**FIGURE 5:**
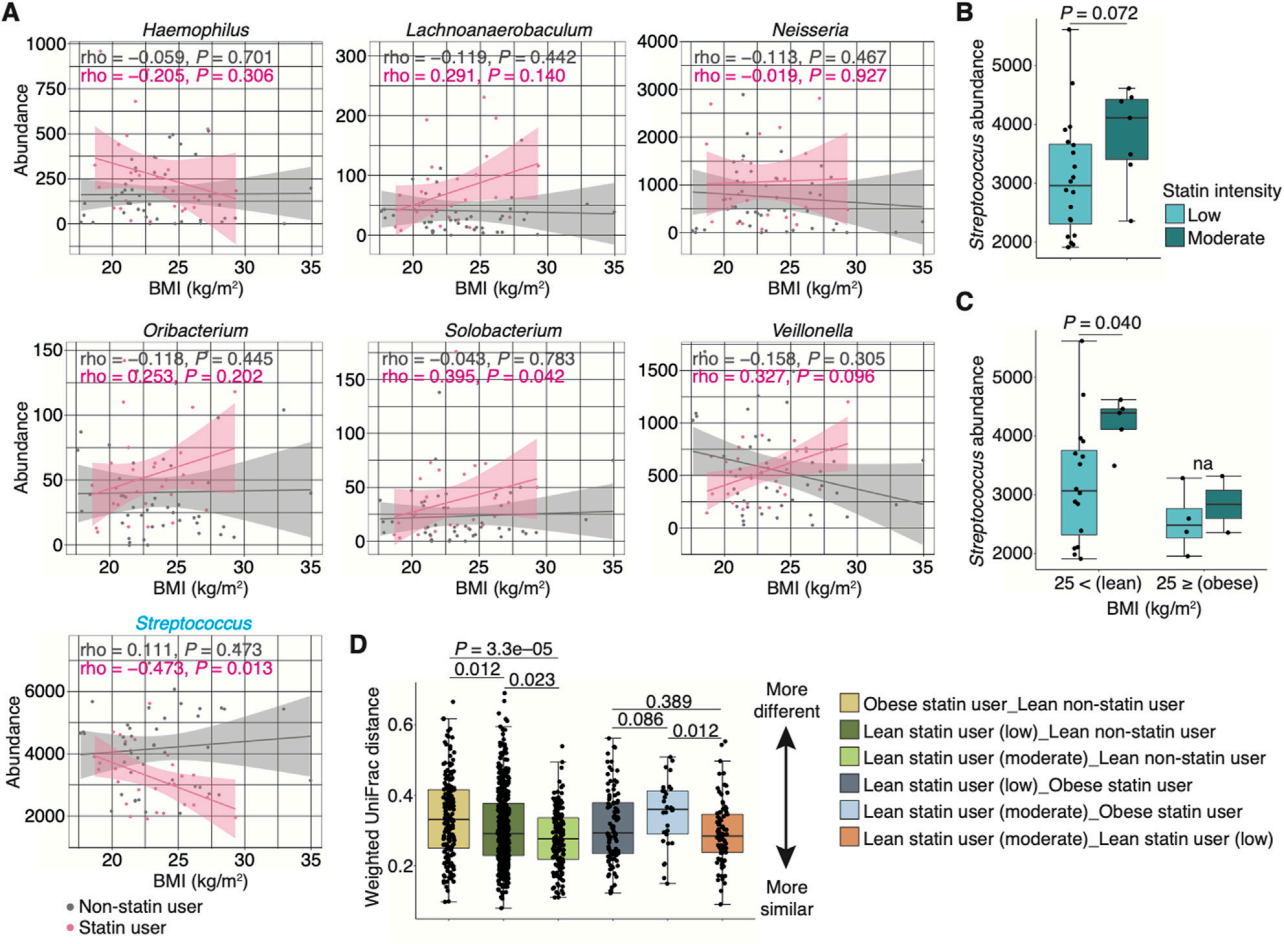
Alteration of the relative *Streptococcus* abundance in statin-users. **(A)** Graphs show the abundance of seven genera that significantly differ between non-statin and statin users (shown in Figure 4C) associated with the BMI in non-statin and statin users (*n* = 44 and 27, respectively). Spearman’s correlation coefficients (i.e., rho) and their *P*-values are depicted in the graph. **(B)** The box plot represents the relative abundance of *Streptococcus* between patients taking low- and moderate-intensity statins (*n* = 27). **(C)** Box plots represent the relative abundance of *Streptococcus* in statin users with BMI below and above 25 with statin intensity (*n* = 27). The pie charts within the figure show the percentage of sex. Statistical significance is determined using the Wilcoxon rank-sum test (*P* < 0.05). Dots represent individual participants. **(D)** Graph shows the median distance between two groups (i.e., lean non-statin users vs. obese statin users, lean statin users taking low-intensity statins, or lean statin users taking moderate-intensity statins, obese statin users vs. lean statin users taking low-intensity statins or those taking moderate-intensity statins, and lean statin users taking low-intensity statins vs. those taking moderate-intensity statins) based on the weighted UniFrac distance. The distribution of users is as follows: lean non-statin users = 30, obese statin users = 6, lean statin users taking low-intensity statins = 16, and lean statin users taking moderate-intensity statins = 5. Statistical significance is determined using the Wilcoxon rank-sum test with the Benjamini–Hochberg method (*P* < 0.05). BMI, body mass index.

The study of the human gut microbiome has shown an association between statin intake and obesity-related microbiome community types based on BMI (Vieira-Silva et al., 2020). Therefore, we first evaluated the correlation between BMI and bacterial abundance (at the genus level) in non-statin and stain users. In seven genera that were significantly different between non-statin and statin users in multivariate analyses, significant negative and positive correlations were observed for *Streptococcus* and *Solobacterium*, respectively, in statin users (rho = −0.473 and 0.395, *P* = 0.013 and 0.042, respectively; Figure 5A). On the contrary, no significant correlations were observed in non-statin users (Figure 5A). We then compared the *Streptococcus* abundance based on statin intensity and BMI. We divided the statin users into two groups based on their BMI according to Japanese standards (Kanazawa et al., 2005): obese (BMI of ≥25) and lean (BMI of <25) statin users. We further classified lean statin users into two categories (i.e., low and moderate intensity) based on the statin intensity. No significant difference in the *Streptococcus* abundance was observed between individuals taking low- and moderate-intensity statins (Figure 5B). Notably, the *Streptococcus* abundance was significantly lower in lean statin users taking low-intensity statins than those taking moderate-intensity statins (*P* = 0.040; Figure 5C). In addition, we evaluated the relative abundance of *Streptococcus* at the species level. No significant changes in the statin intensity were observed in lean statin users (Supplementary Figure S3). Overall, statin intensity and BMI may be key covariates of statin-related alteration of the genus *Streptococcus*.

To compare the dissimilarity in microbiome composition among non-statin users, obese statin, and lean statin users, we evaluated the distance between groups based on the weighted UniFrac distance. We found that the distance between obese statin users and lean non-statin users was the most distant, whereas that between lean statin users taking moderate-intensity statins and lean non-statin users was the closest (*P* < 0.005; Figure 5D). Furthermore, a significant difference was observed between lean statin users taking moderate-intensity statins and obese statin users or lean statin users taking low-intensity statins (Figure 5D). These results suggest that the alteration of salivary microbiome composition is associated with statin intake, intensity, and BMI.

### Functional prediction of the salivary microbiome composition associated with statin use

**FIGURE 6:**
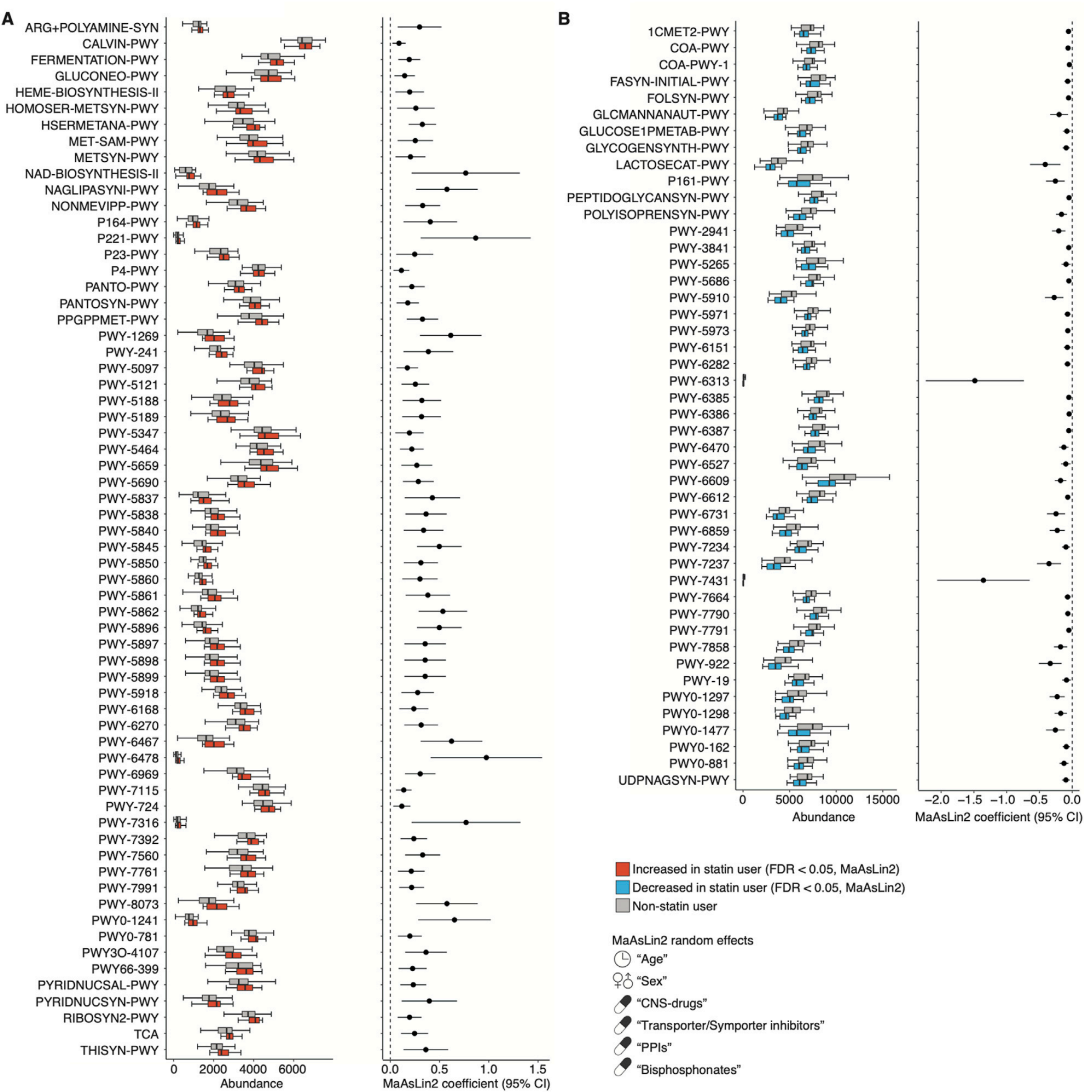
Functional prediction of the salivary microbiome composition associated with statin use. **(A, B)** The left panels show the relative abundance of the representative PICRUSt2-predicted MetaCyc pathway between non-statin and statin users. The right panels show the statistical significance and 95% confidence interval calculated by MaAsLin2 package with age, sex, and the prescribed drugs contributing to the microbiome (CNS drugs, transporter/symporter inhibitors, PPIs, and bisphosphonates) as random effects (FDR <0.05). Confidence interval, CI; CNS, central nervous system; FDR, false discovery rate; PPI; proton pump inhibitor.

**FIGURE 7:**
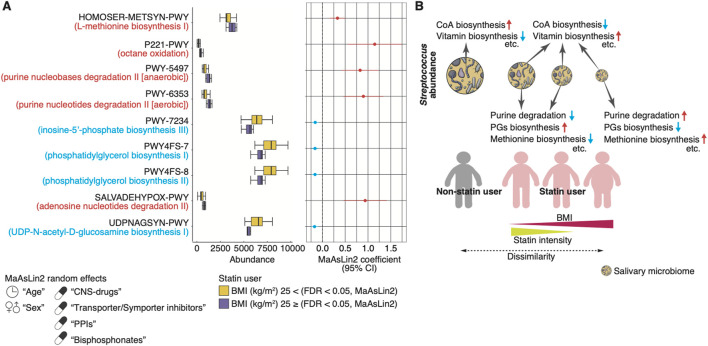
Functional prediction of the salivary microbiome composition associated with obesity in statin users. **(A)** The left panel shows the relative abundance of the representative PICRUSt2-predicted MetaCyc pathway between lean and obese statin users. The right panel shows the statistical significance and 95% confidence interval calculated by MaAsLin2 package with age, sex, and the prescribed drugs contributing to the microbiome (CNS drugs, transporter/symporter inhibitors, PPIs, and bisphosphonates) as random effects (FDR <0.05). **(B)** Schematic illustration of the main results regarding the association of statin intake, intensity, BMI, and salivary microbiome composition changes, including predictive metabolic pathways. In our older adult cohort, we found statin therapy-associated microbiome compositions, including decreasing the *Streptococcus* abundance and changing biosynthesis pathways (e.g., vitamins and CoA). Furthermore, the *Streptococcus* abundance increased in statin users as the BMI decreased and the statin intensity increased, with alteration of predictive metabolic pathways (e.g., purine degradation, PGs biosynthesis, and methionine biosynthesis). The salivary microbiome composition of participants taking moderate-intensity statins is closest to those of lean non-statin users. BMI, body mass index; CoA, coenzyme A; confidence interval, CI, CNS, central nervous system; FDR, false discovery rate; PG, phosphatidylglycerol; PPI, proton pump inhibitor.

To investigate the functional profiles of the microbiome composition associated with statin intake and BMI, we used PICRUSt2 based on the 16S rRNA sequencing data (Douglas et al., 2020). We found that 64 or 46 MetaCyc functional modules were significantly enriched or depleted in statin users compared to non-statin users, respectively, using MaAsLin2 (FDR <0.05; Figure 6; Supplementary Table S4). The enriched modules included the biosynthesis of vitamin K2, B5, and B2 (e.g., PWY-5845, PANTO-PWY, and RIBOSYN2-PWY) and L-methionine (e.g., HSERMETANA-PWY, HOMOSER-METSYN-PWY, and PWY-5347). The depleted modules included the CoA biosynthesis (e.g., COA-PWY and COA-PWY-1). Furthermore, we found that five MetaCyc functional modules related to purine nucleotide degradation and L-methionine biosynthesis were significantly increased in lean statin users compared to obese statin users (MaAsLin2, FDR <0.05; Figure 7A; Supplementary Table S5). A significant decrease in four functional modules, including the phosphatidylglycerol (PG) biosynthesis, was observed in lean statin users compared to obese statin users (MaAsLin2, FDR <0.05; Figure 7A; Supplementary Table S5).

## Discussion

Our main finding showed considerable associations of salivary microbiome alterations with commonly prescribed drugs, including statins, PPIs, transporter/symporter inhibitors, bisphosphonates and CNS drugs, in Japanese older adults with differing comorbidities. Notably, the influence of the drugs was more prominent than that of host variables, including age, sex, and oral health. We also observed a unique alteration of the salivary microbiome, especially the genus *Streptococcus*, associated with statin intake, intensity, and BMI. In order of obese statin users, lean statin users taking low-intensity statins, and lean statin users taking moderate-intensity statins, the *Streptococcus* abundance increased to the comparable abundance of lean non-statin users (Figure 7B). Additionally, functional predictions exhibited significant differences in the biosynthesis (e.g., vitamins, CoA, methionine, and phosphatidylglycerol) and purine nucleotide degradation pathways in statin users (Figure 7B). Our results found an evident relationship among statin intensity, obesity, and microbiome composition changes, suggesting that considering prescription drugs is important for deciphering the relationship between systemic diseases and salivary microbiome changes.

PPIs are a type of acid-suppressive agents that treat gastrointestinal ulcers (Imhann et al., 2016). Previous studies have shown that the human gut microbiome composition is affected by PPI use, and the proportion of oral bacteria is increased in the gut microbiome (Imhann et al., 2016; Jackson et al., 2016). Moreover, the salivary microbiome was shown to be altered in participants who were administered PPIs (Mishiro et al., 2018). Mishiro et al. suggested a relationship between the oral microbiome and PPI use and drug-induced alteration of T helper type 2 (Th2) immunity as an underlying mechanism of this association (Mishiro et al., 2018). Our analyses indicate that statin use may have a greater effect on salivary microbiome compositions than PPI use in older adults. The abundance of several bacteria (i.e., *Lachnoanaerobaculum*, *Oribacterium*, and *Veillonella*) was commonly changed in users taking statin monotherapy and statin/PPI combination therapy. However, no significant difference in the *Strentoroccus* abundance was observed in participants with statin/PPI combination therapy. These results suggest the complex relationship between salivary microbiome alterations and medication in older adults with polypharmacy. Further, bisphosphonates are significantly associated with gut microbial species in patients with osteoporosis (Nagata et al., 2022). The butyrate produced by the gut microbiome is required for the osteoanabolic activity of the parathyroid hormone, which is critical for bone formation (Li et al., 2020). Recently, Lin et al. demonstrated that *Bacteroides vulgatus* in the gut microbiome enhanced inflammation and osteoclast activity by inhibiting valeric acid-producing species, resulting in decreased bone mineral density in human and mice experiments (Lin et al., 2023). Furthermore, bisphosphonates may lead to jaw osteonecrosis, and the underlying pathogenesis involves oral bacterial fluctuations and deficiencies in the host’s innate immune response (Pushalkar et al., 2014; Russmueller et al., 2016).

*Streptococcus* is one of the dominant genera, along with *Veillonella*, *Prevotella*, *Neisseria*, *Haemophilus*, and *Gemella*, in the human salivary microbiome (Nearing et al., 2020; Segata et al., 2012). A Canadian study, which did not consider all prescribed drug usage, showed a relationship between the abundance of salivary microbial species (*Bacteroides*, *Bacillus*, *Catonella*, *Johnsonella*, *Neisseria*, and *Stenotrophomonas*) and statin therapy (DeClercq et al., 2021). However, in our study, the mean abundance of *Bacteroides*, *Bacillus*, *Johnsonella*, and *Stenotrophomonas* was <0.1%, which is considered noise in the sequence. This inconsistency may be associated with interracial, dietary, lifestyle differences, and technical factors (Abdill et al., 2025). In relation to the diseases, the salivary *Streptococcus* is more abundant in individuals with obesity and diabetes compared with individuals without obesity and diabetes (Casarin et al., 2013; Piombino et al., 2014). Obesity is associated with the onset or exacerbation of respiratory diseases, such as asthma (Menegati et al., 2023). The dysbiosis, including increased *Streptococcus pneumoniae*, leads to the dysfunction of T regulatory cells, resulting in more susceptibility to antigens reactions in patients with Th2-related asthma (Menegati et al., 2023). Previous studies have shown that obesity is associated with a longer survival of older adults admitted to the hospital with *S. pneumoniae* infection (Braun et al., 2017; Frasca and McElhaney, 2019). The *Streptococcus* abundance tended to increase in obese non-statin users than in lean non-statin users in our cohort. Interestingly, the *Streptococcus* abundance in statin users was low in obese participants, and their salivary microbiome composition differed most from lean non-statin users (Figure 7B). Along with a decrease in BMI and an increase in statin intensity, the *Streptococcus* abundance increased, and the salivary microbiome composition was closer to lean non-statin users (Figure 7B). Although our multivariate analyses did not consider the therapeutic efficacy, these results suggest that altering the salivary microbiome composition may reflect the host’s physiological changes associated with statin intake and obesity. The discrepancy between obesity and infectious diseases in older adult populations may be due to medications, including statins.

Vieira-Silva et al. demonstrated that statin therapy is a considerable covariate of gut microbiome alteration in patients with obesity (Vieira-Silva et al., 2020). The prevalence of Bacteroides-2 enterotype associated with systemic inflammation gradually increased with elevated BMI in non-statin users. This prevalence is lower in statin users with obesity, showing that obesity-associated gut microbiome dysbiosis was negatively correlated with statin intake (Vieira-Silva et al., 2020). Previous studies showed statin therapy inhibits vitamin K2 production, and their prolonged treatment may decline the vitamin K2 supply to our bodies (Hashimoto and Okuyama, 2017). The depletion of vitamin K is associated with inflammation, especially in patients with cardiovascular diseases (Harshman and Shea, 2016; Yan et al., 2023). On the contrary, the serum vitamin D level may increase in statin users compared to non-statin users (Caglar et al., 2019). Functional predictions revealed that vitamin biosynthesis pathways were increased in statin users, implying a compensatory mechanism to supply the body with depleted vitamins. Furthermore, previous studies showed that PGs are related to inflammation and indirectly regulated by the gut microbiome in obesity (Chen et al., 2021; Kayser et al., 2019; Meikle and Summers, 2017). Purine degradation, which is activated by fructose consumption, is related to obesity, and its metabolites are altered, along with gut dysbiosis, in patients with obesity (Andres-Hernando et al., 2021; Liu et al., 2017; Zhang et al., 2022). Altogether, these studies support that the alteration of salivary microbiome composition in statin users may be related to inflammation and/or immune responses.

Additionally, a significant correlation with longer survival is observed in patients with amyotrophic lateral sclerosis (ALS), a neurodegenerative disease, taking low-intensity statins compared with those not taking statins (Weisskopf et al., 2022). This correlation was not observed in individuals taking high-intensity statins. Previous studies have suggested that statins may attain neuroprotection in CNS (Bagheri et al., 2020; Morimoto et al., 2023; Weisskopf et al., 2022). However, the relationship between statin intake and the incidence of ALS remains unclear (Nabizadeh et al., 2022). Furthermore, Wolosin et al. demonstrated a reduced incidence of dementia and Parkinson’s disease in patients taking statins compared with those taking cardiovascular drugs other than statins (Wolozin et al., 2007). On the other hand, the dysbiosis in the gut and oral microbiome is connected to the onset of ALS in human and mouse experiments (Blacher et al., 2019; Kim et al., 2022; Zeng et al., 2020). The CNS drugs used in this study were anti-depressive agents and anti-parkinsonian drugs, including non-BZD hypnotics, BZD receptor agonists, lithium carbonate, and levodopa/dopa-decarboxylase inhibitors (Supplementary Table S1). The mechanism is unknown, but the salivary microbiome alteration is associated with neurodegenerative diseases and CNS drug use.

This study has some limitations. First, the study has a small number of participants and is limited to a specific region. A large cohort analysis, including cohorts from outside Japan, is needed to assess the true efficacy of prescribed drugs. However, in our cohort, the proportion of prescribed drug usage, such as statins, voltage-gated ion channel-targeting drugs, RAAS-targeting drugs, and PPIs, was similar to those of the global cohorts, including Japan. Furthermore, our results that PPI and bisphosphonate use significantly contributed to the salivary microbiome composition align with previous studies. Although further generalizability analysis is necessary, our results showing statin-associated salivary microbiome changes are applicable in older adult populations. Second, our multivariate and PICRUSt2-predicted MetaCyc pathway analyses are based on 16S rRNA amplicon sequencing, which is a short-read sequencing and limited in its ability to precisely identify bacteria at the species level (Suzuki et al., 2019). Metagenomics sequencing or full-length 16S rRNA amplicon sequencing is necessary for further functional analysis of the salivary microbiome. Third, the influences of the dosing period and therapeutic efficacy were not considered. Nagata et al. reported an association between the duration of drug use and gut microbiome composition at the genus level (Nagata et al., 2022). Generally, the dosing period is closely linked to dosage. Moderate- and high-intensity statins can be prescribed to individuals when low-intensity statins fail to cause a response (Stone et al., 2014). A relationship between statin intensity and prognosis has been observed through the host’s physiological changes. Our finding that the reduction in *Streptococcus* abundance in individuals taking low-intensity than in those taking moderate-intensity implies that the salivary microbiome may be affected by the duration of statin use. Therefore, further longitudinal studies, including dosage and therapeutic efficacy, are needed to elucidate the complex relationship between microbiomes and prescribed drug use. Finally, socioeconomic status and lifestyle factors such as diet were not considered in the analyses. The abundance of several salivary bacteria is independent of the dietary intake but is affected by dietary factors, such as sweeteners (Suez et al., 2022), socioeconomic status and smoking (Belstrøm et al., 2014). Poor socioeconomic status is associated with oral health issues such as periodontitis (Buchwald et al., 2013). Determining the socioeconomic status of the participants is difficult; however, we collected oral health data using questionnaires and showed that oral health did not significantly contribute to the salivary microbiome. However, the accurate assessment of oral health, including conditions such as periodontitis, can be challenging using questionnaires alone. Therefore, obtaining precise information through diagnoses by a professional dentist is required to better understand the relationship between salivary microbiome alterations and prescribed drug use.

In conclusion, we identified a relationship between salivary microbiome alteration and prescribed drug use, particularly statins, in a Japanese older adult population practising polypharmacy. Statin intensity and obesity are associated with changes in the salivary microbiome, including *Streptococcus*, in statin users. Our multivariate analyses, considering prescribed drug use as a confounder, highlight the potential of salivary microbiome profiles as non-invasive biomarkers for accurately monitoring the effects of drug intake and disease progression in older adults.

## Data Availability

Bacterial 16S rRNA sequencing data generated for this study are deposited in https://ddbj.nig.ac.jp/search?query=%22PRJDB17556%22 (accession number: PRJDB17556). Clinical data, except for those within this article, are not available in a public repository to protect the privacy and confidentiality of the study participants. Requests for clinical data can be directed to the corresponding authors and will be reviewed by the Ethics Committee of Juntendo University School of Medicine. All data shared will be de-identified.
