# Supplementary material for: Alteration of salivary Streptococcus is associated with statin therapy in older adults: a cohort study
Source: Front Pharmacol. 2025 Apr 7;16:1455753. doi: 10.3389/fphar.2025.1455753 (PMC12010438; doi:10.3389/fphar.2025.1455753)
Supplement: Supplementary file 1 [file DataSheet1.pdf]

Supplementary Materials

Supplementary tables

Supplementary table 1. Drug category classification of the prescribed drugs based on mode of action.

Supplementary table 2. Explained variance of distance-based redundancy analysis (db-RDA) in 26 drug categories based on the salivary microbiome of the Japanese village volunteer cohort.

Supplementary table 3. MaAsLin2 coefficients of multivariate analyses adjusted for confounders (age, sex, and each prescribed drug) on the salivary microbiome.

Supplementary table 4. MaAsLin2 coefficients, adjusted for confounders (age, sex, CNS drugs, transporter/symporter inhibitors, PPIs, and bisphosphonates), of the PICRUSt2-predicted MetaCyc pathways significantly associated with statin use on the salivary microbiome.

Supplementary table 5. MaAsLin2 coefficients, adjusted for confounders (age, sex, CNS drugs, transporter/symporter inhibitors, PPIs, and bisphosphonates), of the PICRUSt2-predicted MetaCyc pathways significantly associated with obesity on the salivary microbiome of the statin users.

Supplementary figures

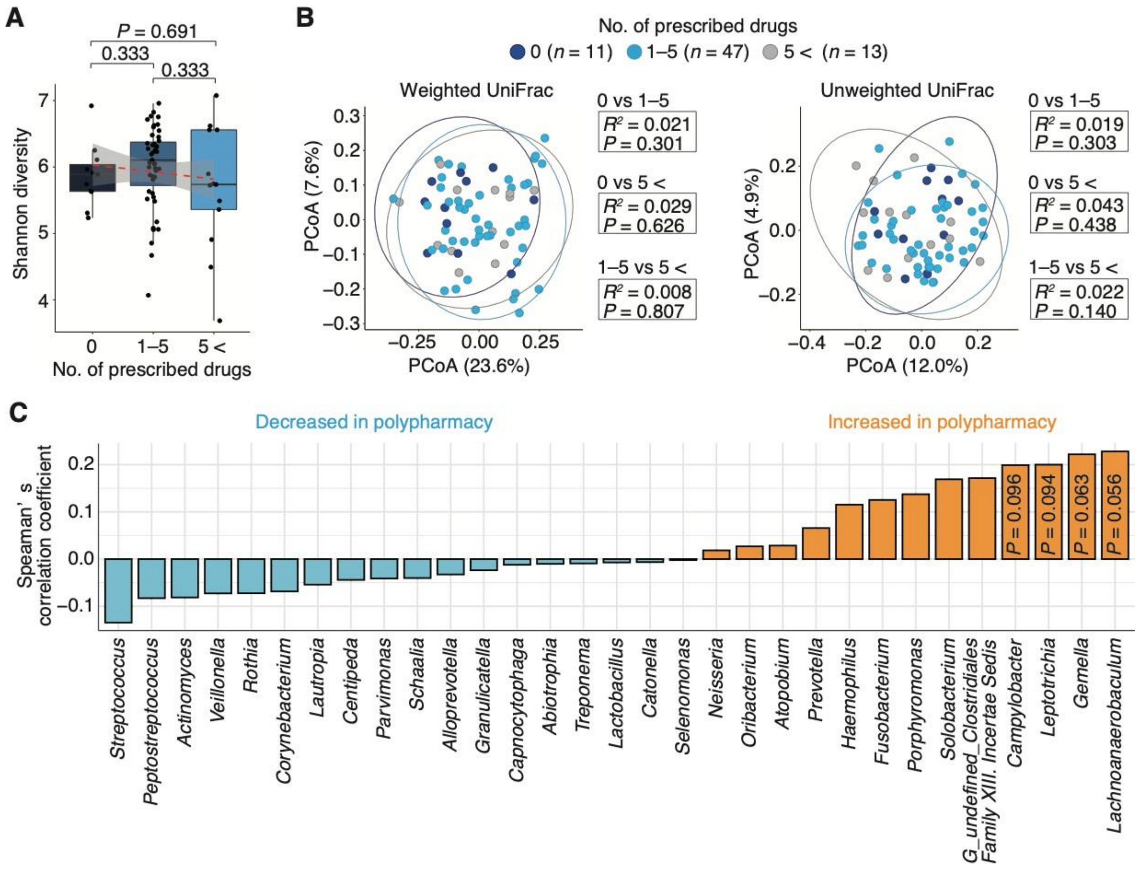

Supplementary figure 1. Salivary microbiome alterations in multiple drug users.

(A) Comparison of the alpha-diversity score (Shannon index) with the number of prescribed drugs in the Japanese village volunteer cohort (*n* = 71). The distribution of participants in three groups dependent on the number of prescription drugs taken is as follows: those taking 0, *n* = 11; those taking 1–5, *n* = 47; those taking ≥6, *n* =13. Red lines and grey areas indicate a regression line and a 95% confidence interval (CI), respectively. Statistical significance was determined using the Wilcoxon rank-sum test with Benjamini–Hochberg correction ($P < 0.05$). **(B)** Weighted and unweighted UniFrac-PCoA in each category based on the number of prescribed drugs (*n* = 71). The number of participants in each category is depicted in the figure. The R^2 and *P*-values were determined using permutational multivariate analysis of variance via the Benjamini–Hochberg method. **(C)** The graph shows the representative genus (mean

abundance of >0.1%) associated with the number of prescribed drugs based on Spearman's correlation coefficients (*n* = 71).

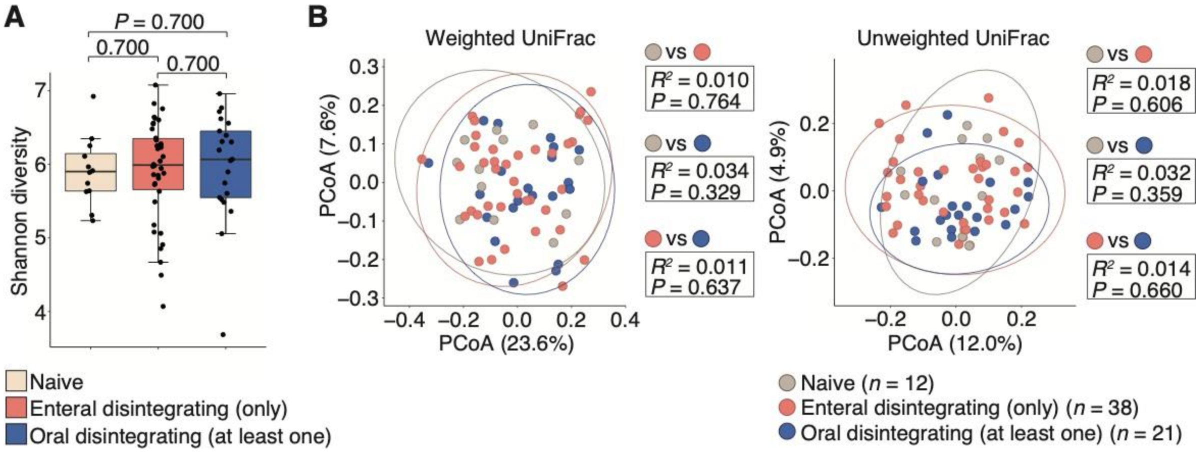

Supplementary figure 2. Salivary microbiome composition and dosage form of prescribed drugs.

(A) Comparison of the alpha-diversity score (Shannon index) in each category in the Japanese village volunteer cohort (*n* = 71). The population was divided into three groups: those taking at least one type of oral disintegrating drug, those taking disintegrating enteral drugs medicines, and treatment-naive participants. Statistical significance was determined using the Wilcoxon rank-sum test with Benjamini–Hochberg correction ($P < 0.05$). Dots represent individual participants. **(B)** Weighted and unweighted UniFrac-PCoA in each category based on the dosage form (*n* = 71). The number of participants in each category is depicted in the figure. The R^2 and *P*-values were determined using permutational multivariate analysis of variance through the Benjamini–Hochberg method.

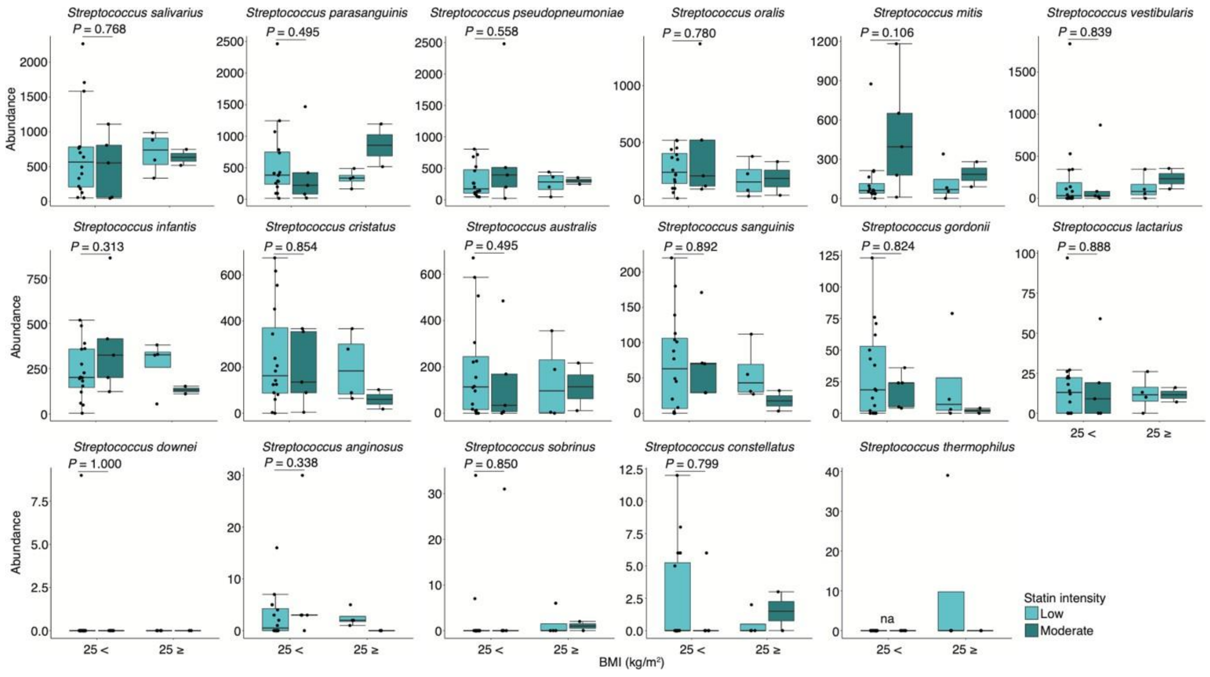

Supplementary figure 3. The relative abundance of *Streptococcus* species in statin-users. Box plots showing the relative abundance of *Streptococcus* species in statin users with statin intensity with BMI < and > 25 (n = 27). Statistical significance was determined using the Wilcoxon rank-sum test (P < 0.05). Dots represent individual participants. na; not available.
